# Pre-Exercise Rehydration Attenuates Central Fatigability during 2-Min Maximum Voluntary Contraction in Hyperthermia

**DOI:** 10.3390/medicina55030066

**Published:** 2019-03-12

**Authors:** Kazys Vadopalas, Aivaras Ratkevičius, Albertas Skurvydas, Saulė Sipavičienė, Marius Brazaitis

**Affiliations:** Department of Applied Biology and Rehabilitation, Lithuanian Sports University, LT-44221 Kaunas, Lithuania; kazys.vadopalas@lsu.lt (K.V.); aivaras.ratkevicius@lsu.lt (A.R.); albertas.skurvydas@lsu.lt (A.S.); saule.sipaviciene@lsu.lt (S.S.)

**Keywords:** dehydration, passive body heating, isometric exercise, sodium chloride

## Abstract

*Background and objectives:* Hyperthermia with dehydration alters several brain structure volumes, mainly by changing plasma osmolality, thus strongly affecting neural functions (cognitive and motor). Here, we aimed to examine whether the prevention of significant dehydration caused by passively induced whole-body hyperthermia attenuates peripheral and/or central fatigability during a sustained 2-min isometric maximal voluntary contraction (MVC). *Materials and Methods:* Ten healthy and physically active adult men (21 ± 1 years of age) performed an isometric MVC of the knee extensors for 2 min (2-min MVC) under control (CON) conditions, after passive lower-body heating that induced severe whole-body hyperthermia (HT, T_re_ > 39 °C) with dehydration (HT-D) and after HT with rehydration (HT-RH). *Results:* In the HT-D trial, the subjects lost 0.94 ± 0.15 kg (1.33% ± 0.13%) of their body weight; in the HT-RH trial, their body weight increased by 0.1 ± 0.42 kg (0.1% ± 0.58%). After lower-body heating, the HT-RH trial (vs. HT-D trial) was accompanied by a significantly lower physiological stress index (6.77 ± 0.98 vs. 7.40 ± 1.46, respectively), heart rate (47.8 ± 9.8 vs. 60.8 ± 13.2 b min^−1^, respectively), and systolic blood pressure (−12.52 ± 5.1 vs. +2.3 ± 6.4, respectively). During 2-min MVC, hyperthermia (HT-D; HT-RH) resulted in greater central fatigability compared with the CON trial. The voluntary activation of exercising muscles was less depressed in the HT-RH trial compared with the HT-D trial. Over the exercise period, electrically (involuntary) induced torque decreased less in the HT-D trial than in the CON and HT-RH trials. *Conclusions:* Our results suggest that pre-exercise rehydration might have the immediate positive effect of reducing physiological thermal strain, thus attenuating central fatigability even when exercise is performed during severe (T_re_ > 39 °C) HT, induced by passive warming of the lower body.

## 1. Introduction

It is well established that whole-body hyperthermia impairs neuromuscular [1,2,3], cognitive performance [4,5], the ability to activate skeletal muscles [1,6], and increases central fatigue during exercise [1,2,7,8]. The results of numerous experiments have proven that work output decreases when the core temperature increases up to a critical point (i.e., rectal temperature > 38.6 °C) [9], especially when intensive activation of the thermoregulatory and cardiovascular systems takes place [10,11,12]. The contractile properties of skeletal muscles are also affected by hyperthermia [13,14]. In hyperthermia, increased contractile speed and rate of muscle relaxation is consistent with peripheral muscle effects, including an increase of sarcoplasmic reticulum (SR) ATPase activity [15], increased muscle fiber conduction velocity [16], increased Ca^2+^ release and uptake rate from the SR, accelerated cross-bridge formation and detachment, and increased actomyosin sensitivity to Ca^2+^ [17]. An increase in muscle contraction and relaxation rate can potentiate muscle force and power [18]. Hyperthermia can have a direct effect on the voluntary activation of skeletal muscles, as the temperature affects the motor unit (MU) firing rate, which is necessary for contraction summation in tetanic contraction [14,19,20]. It has been hypothesized that exercise-induced muscle fatigue under the conditions of hyperthermia may be caused by changes in both the central and the peripheral nervous systems [21]. However, Thomas et al. (2006) provided evidence that hyperthermia can interfere with the function of the central nervous system (CNS) even when there are no measurable effects on the skeletal muscles [13]. There is now strong evidence that a high core temperature promotes central fatigue [3,22]. It appears that, with the increase of the body core temperature up to 38.6 °C, maximal voluntary contraction (MVC) torque decreases, mainly because of changes in the function of the CNS [23]. In fact, passive hyperthermia reduces voluntary activation of exercising muscles during sustained muscle contraction, thus causing greater central fatigability, which is reflected in the decrease of isometric torque production [20,24].

The rate of sweat loss is associated with factors such as exercise intensity, environmental temperature, training status, age, gender, heat acclimation, humidity, and wind speed [25,26,27,28,29]. Dehydration might be one of the factors that acts in addition to heat stress to impair exercise performance during hyperthermia [12]. There is evidence indicating that the brain can undergo structural changes due to plasma osmolality perturbations as a result of heat stress, and the negative impact of heat stress on motor tasks during exercise can be further enhanced by dehydration [30]. Elevated activation of anterior cingulate and superior gyrus, brain areas associated with thirst signaling, speak for enhanced neural activity following dehydration, which is probably associated with greater neural demands for motor tasks and homeostasis maintenance as well [31]. Moreover, hyperthermia with dehydration alters several brain structure volumes, mainly by changes in plasma osmolality, thus strongly affecting neural functions (cognitive and motor) [30]. Hence, there is a question as to whether the prevention of significant dehydration caused by passively induced whole-body hyperthermia attenuates peripheral and/or central fatigability during sustained (2-min) isometric MVC.

Hyperthermia and dehydration are quite common in sports but often occur in combination with other metabolic changes, such as depletion of muscle glycogen and other glucose stores during muscle exercise [32]. However, we hypothesized that hyperthermia without dehydration (HT-RH) and hyperthermia with dehydration (HT-D) can act independently of each other to suppress exercise performance capacity. Passive body heating can be used to study hyperthermia with and without dehydration without confounding effects of the exercise-induced changes in metabolism [23]. Thus, our aim was to study the effects of pre-exercise rehydration on central and peripheral fatigability under conditions of hyperthermia induced by passive lower-body heating.

## 2. Materials and Methods

### 2.1. Volunteers and Experiments

The volunteers were 10 healthy men (age, 21 ± 1 years; height, 174.2 ± 5.3 cm; weight, 70.4 ± 6.5 kg) who were actively engaged in sports (middle-distance runners training ≥10 h per week). The participants were asked to abstain from vigorous exercise and avoid alcohol consumption within 48 h before testing, not to engage in any mental or physical work on the testing day, and not to have any food and drink (except water) within 4 h of testing. The participants slept 7–8 h the night before testing. The study was approved by the Kaunas Regional Ethics Committee of Biomedical Research (Protocol No 130/2005; Authorization No BE-2-54). The volunteers gave informed written consent to take part in the study, which consisted of three separate experiments, namely the control experiment (CON), the hyperthermia with dehydration (HT-D) experiment, and the hyperthermia with rehydration (HT-RH) experiment.

### 2.2. Rationale for the Experiment

The HT-D experiment differed from the CON experiment, in that hyperthermia was induced prior to exercise by applying passive body heating using a heated water bath (44 °C ± 1 °C) [25]. During the HT-RH experiment, hyperthermia was also evoked, but oral rehydration was carried out before exercise. It has been observed that 0.8–1.4 L/h of liquid might be lost by sweating when exercising at a high environmental temperature [32]. The volume of liquid that can be assimilated by physically active volunteers can reach 0.8–1.2 L/h [33]. Hypotonic dehydration and hyponatremia can occur if the loss of isosmotic plasma volume is offset by drinking water [34,35,36]. By sweating, people lose approximately 3.0–4.0 g of NaCl/L [32]. It appears that the amount of lost liquid and NaCl, as well as the circulating blood volume, can be fully restored by performing rehydration using a saline solution (0.9% NaCl), in which the concentration of NaCl is greater than that of sweat [37,38]. In the present experiment, the subjects began drinking the saline solution (0.9% NaCl at 37 °C) 15 min before the start of passive heating, and consumed 100 mL every 6 min. Thus, in the course of 60 min before and during passive heating, the subjects consumed 1000 mL of liquid.

### 2.3. Experimental Protocol

A familiarization session for the MVC evaluation and the electrical stimulation setting were performed on separate occasions. One week later (but not before), the subjects took part in one of the experiments in a random order. The testing was performed at the same time of day. On arrival, the participants voided their bladder, for the estimation of hydration based on urine specific gravity (Arkray Factory Inc. PocketChem UA PU-4010, Kyoto, Japan), and all subjects were found to be well hydrated before all three experiments. After the estimation of hydration levels, the subjects sat still for 30 min at the usual room temperature (22.0 °C ± 0.5 °C) with a relative humidity of 40% ± 0.5%. Subsequently, their heart rate (HR; S-625X, Polar Electro, Kempele, Finland), systolic blood pressure (SBP), and diastolic blood pressure (DBP) were measured using a manual sphygmomanometer (Microlife BP A80, Widnau, Switzerland). The subjects were also weighed, and their rectal and skin temperatures were taken (see Section 2.4). They then performed warm-up exercises, i.e., 10 min of running at a heart rate ranging from 110 to 130 beats/min. Within 10 min after the warm-up period, the subjects were seated in an isokinetic dynamometer chair with their knees fixed at an angle of 120° and rested for 5 min. Subsequently, three 5-s MVC efforts were carried out, with 2-min intervals between them. Before each contraction and 3 s after the contraction, the force generating capacity of the quadriceps muscle was tested by applying a 250 ms stimulation at 100 Hz frequency (TT-100 Hz). After the following 5-min rest, a 2-min MVC was performed with superimposed TT-100 Hz at 3, 14, 29, 44, 59, 74, 89, 104, and 119 s. Every 30 s, the subjects relaxed the exercising muscles for ~3 s and the control TT-100 Hz electrical stimulation was applied. The measurement of resting TT-100 Hz and one 5-s MVC effort with superimposed TT-100 Hz at 15 s (R-15) and 5 min (R-300) was performed after the end of the 2-min MVC. During the exercise, the subject was motivated verbally and received visual information about the force generated.

In the HT-D and HT-RH experimental trials, passive body heating was performed before exercise. Right after the heating, the skin and rectal temperatures were taken (see Section 2.4). No later than 5 min after leaving the bath, the subjects were seated in the dynamometer chair and performed 2-min MVC, followed by recovery. Aiming to reduce the loss of body heat, the subjects wore warm sports clothing and a warm cap (balaclava). At the end of both experiments, the subjects were weighed.

### 2.4. Body Temperature Measurements

Rectal, mean skin, mean body, and inner muscle temperatures were taken before and after passive body heating during the HT-D and HT-RH experiments. Rectal temperature (T_re_) was measured using a probe covered with silicone resin with a built-in thermo-sensing element (Ellab, Rectal probe, Hillerød, Denmark). Before and after passive warming, the subjects placed the probe with a thermo-sensing element into the rectum within 10 s at a depth of 12 cm, as recommended [39]. Skin temperature was measured at three sites on the right side of the body: at the lower part of the scapula (T_1_), in the outward area of the median third of the thigh (T_2_), and the forearm (T_3_). The mean skin temperature (T_sk_) was calculated according to the formula: T_sk_ = 0.5T_1_ + 0.36T_2_ + 0.14T_3_ [40]. The mean body temperature was calculated by applying the formula: T_b_ = 0.65T_re_ + 0.35T_sk_ [41]. The inner muscle temperature was taken with the help of a needle thermometer (Ellab, DM 852, Hillerød, Denmark) at a depth of 3 cm in the lateral part of the quadriceps muscle [42,43].

### 2.5. State of Hydration

The body mass of each participant was assessed by taking their nude mass before and 30 min after the passive heating (TBF-300, Tanita UK, Yiewsley, UK). The state of hydration was calculated using the formula: loss of body mass (%) = (body mass before passive heating − body mass after passive heating)/body mass before passive heating × 100% [44].

### 2.6. Physiological Stress Index

The physiological stress index (PSI) was calculated as follows: PSI = 5 (T_re*t*_ − T_re0_) × (39.5 − T_re0_)^−1^ + (HR*_t_* − HR_0_) × (180 − HR_0_), where T_re0_ and HR_0_ are the calculations performed before the passive body heating, and T_re*t*_ and HR*_t_* are the calculations performed right after the passive body heating [45]. For assessment of PSI, the following scale was used: no stress or extremely low stress (0–2 points); low stress (3–4 points); medium stress (5–6 points); high stress (7–8 points), and extremely high stress (9–10 points).

### 2.7. Subjective Ratings

Thermal sensation and comfort were determined during the passive heating of the body. Every 5 min, the subjects had to assess their thermal sensation on a 10-point scale (neutral, 5 points; unbearably hot, 10 points) and comfort on a 5-point scale (comfortable, 1 point; very uncomfortable, 5 points) [46].

### 2.8. Torque and Electrical Stimulation

The force generating capacity of the knee extensor muscles was measured using the isokinetic dynamometer (System 3; Biodex Medical Systems, Shirley, New York, NY, USA). Volunteers sat upright with the knee joint positioned at a 120° angle (a 180° angle is full knee extension). Muscle surface electrical stimulation was applied using two carbonized rubber electrodes covered with an electrode gel (ECGEEG Gel; Medigel, Modi’in, Israel). One of the electrodes (6 × 11 cm) covered the proximal portion of the quadriceps femoris. Another electrode (6 × 20 cm) covered the distal part of the muscle, above the patella. The electrical stimulation was delivered using a standard electrical stimulator (MG 440; Medicor, Budapest, Hungary) in 1-ms square-wave pulses.

### 2.9. Central Activation Ratio Measurement

The central activation ratio (CAR) was assessed to quantify muscle voluntary activation. CAR is considered as the direct index of muscle activation by the CNS. It is based on the difference in contraction force between MVC and MVC with superimposed TT-100 Hz., i.e., CAR% = MVC/ (MVC + TT-100 Hz) × 100 [47,48].

### 2.10. Statistical Analysis

The interval data were tested for normal distribution using the Kolmogorov–Smirnov test, and all interval data were found to be normally distributed. Descriptive data are presented as the mean ± standard deviation (SD). Single time-point comparisons between visits were analyzed using dependent-sample t tests for the baseline values. One-way ANOVA for repeated measures was used to determine the effects of HT-D vs. HT-RH experimental trials of two levels on changes in body temperature, cardiovascular response, loss in body weight, and PSI values. Two-way ANOVA for repeated measures was used to determine the effects of experimental trials of three levels (CON vs. HT-D vs. HT-RH) × time of 10 levels (during 2-min MVC) or three levels (post-exercise recovery) on MVC and CAR; and time of five levels (during 2-min MVC) or three levels (post-exercise recovery) on electrically induced muscle contractility properties (TT-100 Hz).

A two-way ANOVA for repeated measures was also performed to study the effects of pre-exercise rehydration with hyperthermia of two levels (HT-D vs. HT-RH) × time of two levels (before vs. after lower-body heating) on body temperature, cardiovascular response, and body weight.

If significant effects were established, Sidak’s post hoc adjustment was used for multiple comparisons within each repeated-measure ANOVA. Observed power (OP, %) was calculated, and the partial eta squared (*η_p_*^2^) was estimated as a measure of the experimental condition effect size. The nonparametric Wilcoxon signed-rank test for two related samples was used as ordinal data to compare changes in subjective perception (thermal and comfort sensations). Data were analyzed using SPSS version 21.0 (IBM Corp., Armonk, NY, USA).

## 3. Results

There was no difference between the CON, HT-D, and HT-RH experiments in all the initial (baseline) variables measured before exercise and at 3-s time point of 2-min MVC (trial effect, *p* > 0.05).

Passive lower-body heating resulted in significant increases in body temperature (T_re_, T_sk_, T_b_, and T_mu_) in both the HT-D and HT-RH trials (time effect; *p* < 0.001, *η_p_*^2^ > 0.7, OP > 99%), with no time × trial interaction (Table 1). The PSI increased less in the HT-RH trial than in the HT-D trial (trial effect; *p* < 0.05, *η_p_*^2^ = 0.35, OP = 87%). In the HT-D trial, the subjects lost 0.94 ± 0.15 kg (1.33% ± 0.13%) of their body weight; in the HT-RH trial, their body weight increased by 0.1 ± 0.42 kg (0.1% ± 0.58%) (trial effect; *p* < 0.001, *η_p_*^2^ = 0.85, OP = 100%). The HR increased more in the HT-D trial than in the HT-RH trial (60.8 vs. 47.8 b min^−1^, respectively) (trial effect; *p* < 0.001, *η_p_*^2^ = 0.61, OP = 97%). In the two experimental trials, DBP decreased significantly (time effect; *p* < 0.01, *η_p_*^2^ = 0.42, OP = 83%), with no time × trial interaction. The SBP decreased only in the HT-RH trial, with a significant time × trial interaction (*p* < 0.05, *η_p_*^2^ = 0.29, OP = 81%).

**Figure 1:**
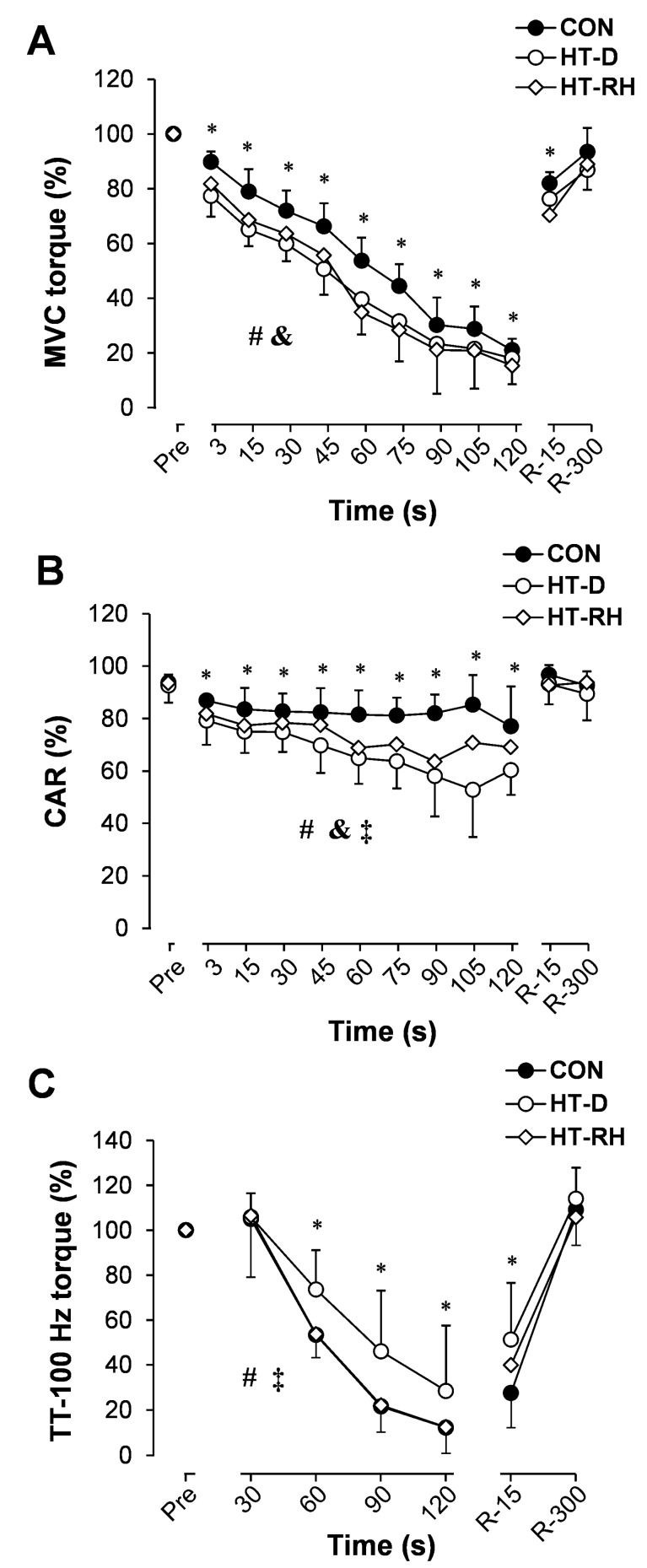
Maximal voluntary contraction (MVC) torques (**A**), central activation ratio (CAR) (**B**), and TT-100 Hz torque (**C**) during 2-min MVC, followed by recovery 15 s (R-15) and 300 s (R-300) after the exercise in the control (CON) experiment and in experiments with hyperthermia with dehydration (HT-D) and hyperthermia with rehydration (HT-RH). The values pertaining to the pre-exercise period (Pre) were taken from the initial measurements performed in these experiments. * *p* < 0.05 compared with Pre values; # *p* < 0.05 CON compared with HT-D; ‡ *p* < 0.05 CON compared with HT-RH; and & *p* < 0.05 HT-D compared with HT-RH.

As shown in Figure 1A, MVC torque decreased more in the HT-D and HT-RH trials (time effect; *p* < 0.001, *η_p_*^2^ = 0.90, OP = 100%) than in the CON trial (trial effect; *p* < 0.05, *η_p_*^2^ = 0.32, OP = 92%) during exercise (2-min MVC), with no time × trial interaction. In the HT-D trial, TT-100 Hz torque decreased less (time effect; *p* < 0.001, *η_p_*^2^ = 0.90, OP = 100%) than in the CON and HT-RH trials (trial effect; *p* < 0.05, *η_p_*^2^ > 0.3, OP > 90%) during 2-min MVC, with a significant time × trial interaction (*p* < 0.01, *η_p_*^2^ = 0.32, OP = 80%) (Figure 1C). During recovery, MVC and TT-100 Hz torque returned to the pre-exercise value 5 min (R-300) after the exercise, with no time × trial interaction.

The voluntary activation (CAR) of exercising muscles decreased over the exercise period in all three experimental trials (time effect; *p* < 0.001, *η_p_*^2^ = 0.62, OP = 98%) (Figure 1B). The results of the present study indicate a smaller CAR decrease during exercise in the HT-RH trial vs. the HT-D trial (trial effect; *p* < 0.05, *η_p_*^2^ = 0.32, OP = 82%), with a significant time × trial interaction (*p* < 0.01, *η_p_*^2^ = 0.42, OP = 86%). Moreover, 15 s (R-15) after 2-min MVC, the CAR indices returned to their initial values (before exercise), with no time × trial interaction.

In the HT-D trial, the thermal sensation increased from “slightly warm” (6 points) to “hot” (8 points) (*p* < 0.01) and thermal comfort shifted from “comfortable” (1 point) to “uncomfortable” (3 points) (*p* < 0.01). In the HT-RH trial, thermal sensation increased from “slightly warm” (6 points) to “warm” (7 points) (*p* < 0.05), whereas comfort shifted from “comfortable” (1 point) to “slightly uncomfortable” (2 points) (*p* < 0.05). These changes did not differ significantly between the HT-D and HT-RH experimental trials (*p* > 0.05).

## 4. Discussion

To our knowledge, we are the first to investigate effects of water replacement on the central and peripheral fatigue during the continuous 2 min MVC in the state of passively induced whole body hyperthermia. As expected, in the present study, we showed that rehydration was a sufficient factor to decrease physiological (PSI) and cardiovascular (HR and blood pressure) thermal strain in whole-body hyperthermia. Although the lower thermal strain of HT-RH was insufficient to affect body temperature and subjective perception compared with HT-D, central fatigability was attenuated during the 2-min MVC. In the HT-RH trial (vs. the HT-D trial), the greater inhibition of the force-generating capacity was accompanied by a greater voluntary activation of the exercising muscle during the continuous MVC in the HT-RH trial, compared to the HT-D trial.

A failure to sustain voluntary activation of exercising muscles under the maximal effort indicates that the neural drive to the muscle is less than optimal [49], whereas peripheral fatigue is associated with processes within the muscle and includes excitation–contraction coupling and inhibition of cross bridge cycling by metabolites [50,51]. Both central and peripheral mechanisms can contribute to muscle force loss, and discrimination between these mechanisms is often complicated [52,53]. The electrical stimulation (TT-100 Hz) superimposed on the voluntary contraction was used to distinguish between central and peripheral mechanisms during continuous 2-min MVC [53,54]. In agreement with Racinais et al. (2008), Brazaitis et al. (2010), and Todd et al. (2005), our data shows that passive hyperthermia induces an additional decrease in voluntary activation (i.e., greater central fatigue) [1,8,17]. This additional decrease is not due to interference with the peripheral transmission of the neural drive and is likely to be associated with the supraspinal failure [1]. In comparison with a neutral environment, hyperthermia causes reduction in the amplitude of superimposed twitches generated by motor cortex stimulation during continuous MVC [14].

Water, hormonal, and neurotransmitter balances are disturbed in the heat-stressed brain. Brain morphology is transformed by dehydration in hyperthermia and has been shown to have a negative effect on cognitive–motor performance [30]. A body water deficit stresses the brain and elicits greater neural demands during cognitive–motor tasks and may accelerate fatigability [55,56]. Water replacement in the heat can attenuate reduction in the circulation in the brain, kidneys, and skin, and thus attenuate adverse effects of overheating [37,38]. In the present study, we investigated whether the prevention of significant dehydration caused by passively induced whole-body hyperthermia attenuates peripheral and/or central fatigability during sustained (2-min) isometric MVC. As expected, pre-exercise rehydration (HT-RH) decreased physiological and cardiovascular thermal strain sufficiently and attenuated central fatigability during prolonged exercise in hyperthermia. In general, greater voluntary activation of exercising muscles during continuous contractions may lead to greater metabolite accumulation and ATP depletion, slowed Ca^2+^ kinetics, a reduction in actomyosin sensitivity to Ca^2+^, and impaired cross-bridge cycling [17]. As a result, mainly because of hyperthermia-induced changes in the periphery, such as an increase in the speed of muscle contraction and relaxation [2,57], greater voluntary activation in HT-RH stressed the muscle and decreased electrically (TT-100 Hz) induced torque production to a greater extent compared with HT-D during the 2-min MVC.

The morphological differences between men and women [58] and the deterioration in thermoregulatory responses to extreme temperatures in older individuals [15,59] suggest that the results of the present study on young men cannot be generalized to women and other age groups. Another possible limitation is that we could not control precisely the balance of rehydration-to-dehydration levels, mainly because of methodological considerations (e.g., heat acclimation effect).

Sports drinks often contain salts to replace the loss of electrolytes during exercise, but the concentration of sodium, chloride, and potassium ions in these drinks is usually significantly lower than in sweat [60]. It has been suggested that endurance athletes competing in hot environments and those with salty sweat or an inability to properly match liquid intake with sweat loss would benefit from salt supplements in addition to sports drinks [61]. In addition to this, it has been shown that a delayed effect of oral 0.9% or 1.07% saline ingestion on plasma volume, blood, and urine osmolality, which in the case of 0.9% saline fluid peaks 1–2 h after ingestion (1 L within 30 min) was completed [62]. Based on reported plasma volume and osmolality kinetics, it seems reasonably indicative that in our case, at the moment of exercise (2-min MVC), the plasma osmolality was restored close to homeostatic level and were not much below or above the physiological level. It is novel that fluid replacement by using oral 0.9% saline ingestion is an effective way for reducing physiological and cardiovascular thermal strain and attenuating central fatigability when exercise is performed during severe whole-body hyperthermia. Thus, for practitioners, it is advantageous to use salt supplements to compensate for the loss of electrolytes in long-lasting endurance events and to prevent central fatigability.

## 5. Conclusions

Our results suggest that pre-exercise rehydration might have an immediate positive effect in reducing physiological and cardiovascular thermal strain, thus attenuating central fatigability, even when exercise is performed during severe (T_re_ > 39 °C) hyperthermia induced by passive warming of the lower body.

## Figures and Tables

**Table 1:** Body temperature, body weight, heart rate, blood pressure, and thermal strain before and after lower-body heating in experiments of hyperthermia with dehydration (HT-D) and hyperthermia with rehydration (HT-RH).

	HT-D		HT-RH	
	Baseline	End-Heating	Baseline	End-Heating
T_re_, °C	37.38 ± 0.23	39.36 ± 0.30 *	37.22 ± 0.23	39.32 ± 0.48 *
T_b_, °C	36.34 ± 0.21	38.45 ± 0.33 *	36.49 ± 0.24	38.63 ± 0.24 *
T_sk_, °C	34.42 ± 0.55	36.77 ± 0.66 *	34.61 ± 0.85	36.95 ± 0.59 *
T_mu_, °C	36.97 ± 0.28	39.96 ± 0.31 *	36.91 ± 0.29	39.83 ± 0.31 *
Body weight, kg	70.42 ± 6.54	69.48 ± 6.42 *	71.06 ± 7.11	70.96 ± 6.78
HR, b min^−1^	69.00 ± 5.57	129.80 ± 23.45 *	66.20 ± 7.29	114.00 ± 13.87 *^,&^
SBP, mmHg	127.00 ± 18.61	129.00 ± 26.66	131.20 ± 13.85	120.60 ± 7.70 *^,&^
DBP, mmHg	79.40 ± 13.18	62.00 ± 19.40 *	74.60 ± 5.37	58.40 ± 9.24 *
PSI		7.40 ± 1.46		6.77 ± 0.98 ^&^

T_re_, rectal temperature; T_b_, mean body temperature; T_sk_, mean skin temperature; T_mu_, intramuscular temperature; HR, heart rate; SBP, systolic blood pressure; DBP, diastolic blood pressure; PSI, physiological stress index. Values are shown as the mean ± SD. * *p* < 0.05 compared with the baseline and ^&^ *p* < 0.05 compared with HT-D.
